# Comprehensive analysis of PM20D1 QTL in Alzheimer’s disease

**DOI:** 10.1186/s13148-020-0814-y

**Published:** 2020-02-03

**Authors:** Jose Vicente Sanchez-Mut, Liliane Glauser, David Monk, Johannes Gräff

**Affiliations:** 1 Laboratory of Neuroepigenetics, Brain Mind Institute, School of Life Sciences, Ecole Polytechnique Fédérale de Lausanne, CH-1015 Lausanne, Switzerland; 2 Genomic Imprinting Cancer Group, Institut d’Investigació Biomedica de Bellvitge, E-08908 Barcelona, Spain; 3 Biomedical Research Centre, School of Biological Sciences, University of East Anglia, NR4 7TJ Norwich, UK

**Keywords:** Alzheimer, mQTL, eQTL, QTL, DNA methylation, RNA expression, PM20D1, SLC41A1, NUCKS1, RAB7L1

## Abstract

**Background:**

Alzheimer’s disease (AD) is a complex disorder caused by a combination of genetic and non-genetic risk factors. In addition, an increasing evidence suggests that epigenetic mechanisms also accompany AD. Genetic and epigenetic factors are not independent, but multiple loci show genetic-epigenetic interactions, the so-called quantitative trait loci (QTLs). Recently, we identified the first QTL association with AD, namely *Peptidase M20 Domain Containing 1* (*PM20D1*). We observed that *PM20D1* DNA methylation, RNA expression, and genetic background are correlated and, in turn, associated with AD. We provided mechanistic insights for these correlations and had shown that by genetically increasing and decreasing PM20D1 levels, AD-related pathologies were decreased and accelerated, respectively. However, since the PM20D1 QTL region encompasses also other genes, namely *Nuclear Casein Kinase and Cyclin Dependent Kinase Substrate 1* (*NUCKS1*); *RAB7*, *member RAS oncogene family-like 1* (*RAB7L1*); and *Solute Carrier Family 41 Member 1* (*SLC41A1*), we investigated whether these genes might also contribute to the described AD association.

**Results:**

Here, we report a comprehensive analysis of these QTL genes using a repertoire of in silico methods as well as in vivo and in vitro experimental approaches. First, we analyzed publicly available databases to pinpoint the major QTL correlations. Then, we validated these correlations using a well-characterized set of samples and locus-specific approaches—i.e., Sanger sequencing for the genotype, cloning/sequencing and pyrosequencing for the DNA methylation, and allele-specific and real-time PCR for the RNA expression. Finally, we defined the functional relevance of the observed alterations in the context of AD in vitro*.* Using this approach, we show that only *PM20D1* DNA methylation and expression are significantly correlated with the AD-risk associated background. We find that the expression of *SLC41A1* and *PM20D1*—but not *NUCKS1* and *RAB7L1*—is increased in mouse models and human samples of AD, respectively. However, *SLC41A1* and *PM20D1* are differentially regulated by AD-related stressors, with only *PM20D1* being upregulated by amyloid-β and reactive oxygen species, and with only *PM20D1* being neuroprotective when overexpressed in cell and primary cultures.

**Conclusions:**

Our findings reinforce *PM20D1* as the most likely gene responsible of the previously reported *PM20D1* QTL association with AD.

## Background

Alzheimer’s disease (AD) is the most common neurodegenerative disorder in western societies. It is characterized by a progressive decline in mental abilities, neuronal loss, and the accumulation of two types of protein aggregates, amyloid plaques and neurofibrillary tangles [1]. The causes of AD remain elusive, but AD occurrence is currently understood as the consequence of a complex combination of genetic and non-genetic factors [2], the latter of which are believed to be mediated by epigenetic mechanisms [3, 4].

The genetic component of AD has been generally interrogated by genome-wide association studies (GWAS), which have identified an important number of risk loci associated with AD [5–7], but a causal relationship thereof remains difficult to establish. In contrast, the epigenetic contribution to AD is mainly interrogated by locus-specific or epigenome-wide association studies (EWAS) [8, 9], which have revealed site-specific epigenetic alterations and thereby provide mechanistic insights for a particular risk gene, but often lack the statistical power of GWAS [10]. Combining both approaches, it is now possible to identify single nucleotide polymorphisms (SNPs) that correlate with alterations in DNA methylation levels—the so-called methylation quantitative trait loci (mQTLs).

Recently, we reported the first mQTL association with AD, which converges on the gene *Predicted Metalloproteinase 20D1* (*PM20D1*) [11]. We have shown that AD-related cellular stressors—such as the presence of reactive oxygen species (ROS) and amyloid-beta (Aβ)—increase *PM20D1* expression, that *PM20D1* expression is upregulated in symptomatic APP/PS1 AD mice and human AD samples, and that genetic manipulation of PM20D1 levels can modify the progression of the disease in APP/PS1 mice: When PM20D1 was overexpressed, disease progression was delayed; when PM20D1 was decreased, disease progression was accelerated.

At the same time, two high-throughput studies have expanded the *PM20D1* expression QTL (eQTL) region, showing that the expression of neighboring genes to *PM20D1* also correlate with several SNPs in that region, which include *Nuclear Casein Kinase And Cyclin Dependent Kinase Substrate 1* (*NUCKS1*); *RAB7*, *member RAS oncogene family-like 1* (*RAB7L1*); and *Solute Carrier Family 41 Member 1* (*SLC41A1*), plus *PM20D1* [12, 13]. Therefore, in spite of the reported functional validations for *PM20D1* [11], we cannot completely exclude the possibility that these genes also contribute to the progression of AD.

In the present study, we combine a series of in silico methods with in vivo and vitro experiments to provide a comprehensive analysis of *PM20D1* eQTL genes, curated evidence for an AD association centered on *PM20D1*, and further support for the protective role of PM20D1 in AD.

## Methods

### DNA methylation and genetic background

Hannon et al.’s mQTL database was interrogated for mQTLs—SNPs and CpGs—in each of the potential PM20D1 QTL region genes [14]. When significant, Bonferroni-corrected *p* values were directly reported. mQTL SNPs were then investigated in our own postmortem human brain cohort of samples from the IDIBELL Biobank (Barcelona, Spain). DNA was isolated by phenol-chloroform extraction from gray matter of 18 control (Braak 0–II; 32% female; age 64 ± 3 years, mean ± SEM) and 21 Alzheimer’s disease *frontal cortex* samples (Braak V–VI; 43% female; age 77 ± 2 years, mean ± SEM). Genotypes were obtained by Sanger sequencing using primers listed in Additional file 1: Table S1. DNA was bisulfite converted using the EZ DNA methylation kit (Zymo Research), and tested for bisulfite cloning/sequencing and pyrosequencing as previously described [11]. Bisulfite conversion was ensured by including non-CG cytosines in the dispensation sequence following the manufacturer’s instructions. Primers for bisulfite cloning/sequencing and pyrosequencing are listed in Additional file 1: Table S1.

### RNA expression and genetic background

GTEX [12] and LIBD [13] datasets were investigated for eQTL correlations in each of the potential PM20D1 QTL region genes. When significant, FDR-corrected *p* values were directly reported. Only previously annotated genes were considered for LIBD prefrontal (PFC) and hippocampal (Hip) datasets—i.e., Type = “Gene,” Class = “InEns”; and Type = all, Class = “InGen,” respectively. eQTL SNPs were then investigated in our cohort of samples. RNA purification was performed using TRIzol (Invitrogen), reverse-transcribed using the Thermoscript RT-PCR system (Invitrogen), and tested using StepOnePlus Real-Time PCR System (Applied Biosystems) and SYBR Green PCR MasterMaster Mix (Applied Biosystems). Three housekeeping genes were used for normalizing PCR signals. Primers for real-time PCR are listed in Additional file 1: Table S1.

The GeneNetwork database (http://www.genenetwork.org) was also analyzed for the PM20D1 QTL region genes in the BxD mice population. BxD mice derive from multiple intercrosses of the C57BL/6 J (B) and DBA/2 J (D) progenitor mice, later inbred to fix the generated genetic variation [15]. To date, close to 200 BxD strains have been generated and extensively characterized—at genetic, transcriptomic, and phenotypic levels (http://www.genenetwork.org/)—which constitutes a well-established genetic reference for the analysis of QTLs [16]. The eQTL analysis was performed using the BxD recombinant inbred (RI) Family group, Liver mRNA type, EPFL/LISP BXD CD Liver Affy Mouse Gene 1.0 ST (Apr13) RMA Exon Level dataset, with the interval mapping for the entire genome [16]. Images from GeneNetwork Map Viewer are represented.

### QTL expression in Alzheimer’s disease

PM20D1 QTL region gene expression was investigated in the aforementioned human brain cohort and five 12-month-old APP/PSEN1 [17] and five wild-type littermate male mouse frontal cortex samples. All animals were maintained under standard animal house conditions in a 12-h dark-light cycle with free access to food and water. The experimental procedures were conducted according to EPFL’s and Switzerland’s guidelines on animal welfare (cantonal animal experimentation authorization numbers VD2875.1 and VD3169).

### Functional validation

SH-SY5Y neuroblastoma cells (ATCC) were cultured in DMEM supplemented with 20% FBS, 100 μ/ml penicillin, and 100 mg/ml streptomycin at 37 °C in a humidified atmosphere of 5% CO_2_. SH-SY5Y cells were treated with 0.2‰ hydrogen peroxide (Merck) and with synthetic amyloid-β (1–42)-derived diffusible ligands (ADDLs) (Abcam) during 6 h and 24 h. Cell viability was measured using Alamarblue cell viability assay (Invitrogen) according to the manufacturer’s instructions. Primary hippocampal neuron-glia co-cultures derived from P0 wild-type mice were cultured in media consisting of Neurobasal (Invitrogen), B27 supplement (Invitrogen), l-glutamine (Invitrogen), and penicillin/streptomycin (Invitrogen) (0.2 ml per well) on 96-well plates (2.5 × 10^4^ cells per well) coated with Cultrex poly-l-lysine (Trevigen). Cells were infected at DIV6 with 20 × 10^3^ (200 ng/well) viral particles containing either a GFP (mock) or a PM20D1/SLC41A1 version of the pLVX-IRES-ZsGreen1vector (Promega). At DIV 14–17, cytotoxicity was assessed using the CytoTox96 non-radioactive cytotoxicity assay (PROMEGA) according to the manufacturer’s instructions.

### Statistical analysis

The analyses were performed using Prism 6.0 (GraphPad). Correlations were calculated using Pearson’s correlation coefficients, and comparisons using one-way ANOVA with post hoc Holm-Sidak’s multiple comparison tests. *p* values smaller than 0.05 were considered statistically significant and provided in the figures as follows: *p < 0.05, **p < 0.01, ***p < 0.005, and ****p < 0.0001.

## Results

### DNA methylation and genetic background

**Fig. 1:**
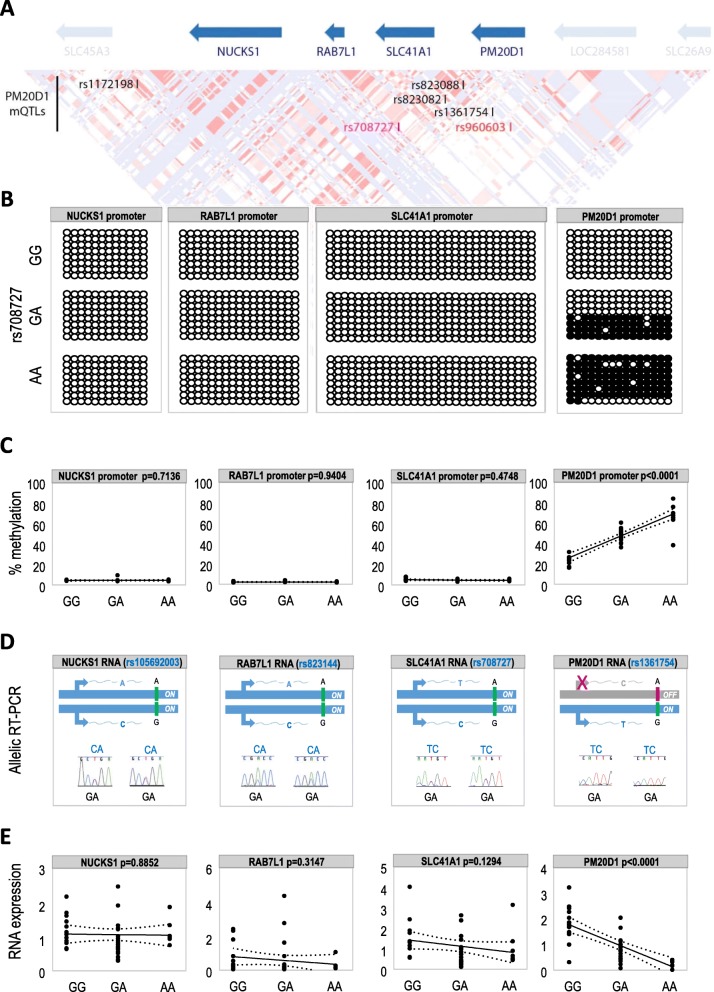
Overview of the full *PM20D1* QTL region and its relation to AD in human frontal cortex. **a** The region comprises several genes (blue arrows) and is in partial linkage disequilibrium (HapMap CEU recombination map is indicated below the genes). *PM20D1* mQTLs are indicated in black, of which the mQTLs most strongly associated with AD in bold magenta (rs708727) and bold red (rs960603) [11]. **b** Locus-specific bisulfite sequencing of *PM20D1* QTL region genes. **c** rs708727 correlations with the levels of DNA methylation of *PM20D1* QTL genes in human frontal cortex measured by pyrosequencing. **d** rs105692003, rs823144, rs708727, and rs1361754 are located in the coding region of *NUCKS1*, *RAB7L1*, *SLC41A1*, and *PM20D1*, respectively, and in linkage disequilibrium. Retrotranscription PCR Sanger sequencing of heterozygous samples detects RNA transcripts from both chromosomes for all genes, except for *PM20D1* in which only one of the chromosomes is active. **e** rs708727 correlates with the levels of RNA expression of *PM20D1* QTL genes in human frontal cortex measured by qRT-PCR. Single values are represented by dots, *p* values from Pearson’s correlations

*PM20D1* DNA methylation is strongly correlated with the rs708727-rs960603 haplotype, and both, methylation and haplotype, are associated with AD [11] (Fig. 1a). In particular, multiple CpG sites in the *PM20D1* promoter show strong correlations with rs1172198, rs708727, rs823082, rs823088, rs1361754, and rs960603 mQTL SNPs [14] (Table 1). Our previous whole-genome bisulfite sequencing (WGBS) analysis discarded similar correlations with PM20D1 neighboring genes [11]. However, since the eQTL region has been recently expanded in other tissues, which now also include the genes *NUCKS1*, *RAB7L1*, and *SLC41A1* [12, 13] (Table 2), we cannot completely rule out the possibility that these genes show similar correlations. In particular, the GTEX consortium [12] found correlations between these particular mQTLs and the levels of RNA expression for *NUCKS1*, *RAB7L1*, and *SLC41A1* in the cerebellum; for *PM20D1* in the hippocampus; and for *NUCKS1*, *RAB7L1*, *SLC41A1*, and *PM20D1* in other non-brain tissues (e.g., blood, tibial nerve) [12]. In addition, the LIBD study [13] found correlations for *RAB7L1*, *SLC41A1*, and *PM20D1* in the dorsolateral prefrontal cortex, and for *RAB7L1* and *PM20D1* in the hippocampus [13] (Table 2).

**Table 1:** *PM20D1* mQTL region genes and SNPs and their DNA methylation correlation obtained from the Hannon database

	CpG	rs1172198	rs708727	rs823082	rs823088	rs1361754	rs960603	Min	Max	Delta
NUCKS1	-	-	-	-	-	-	-	-	-	-
RAB7L1	-	-	-	-	-	-	-	-	-	-
SLC41A1	cg23034840	-	7.60E−08	-	2.90E−05	-	-	7	9	2
PM20D1	cg07157834	-	3.60E−22	-	-	-	-	83	90	7
PM20D1	cg24503407	4.60E−08	1.90E−25	7.20E−16	-	7.20E−16	1.80E−12	60	87	27
PM20D1	cg07167872	2.80E−07	5.00E−26	7.00E−16	4.10E−10	6.90E−16	1.90E−13	41	79	38
PM20D1	cg11965913	2.30E−11	2.30E−37	2.50E−19	1.50E−10	2.50E−19	1.30E−14	15	75	60
PM20D1	cg14893161	1.30E−12	4.30E−34	7.00E−19	1.30E−09	7.00E−19	1.40E−13	17	70	53
PM20D1	cg14159672	1.30E−08	3.00E−30	3.20E−18	1.20E−10	3.20E−18	3.10E−13	22	87	65
PM20D1	cg26354017	1.40E−09	5.60E−33	2.10E−18	4.50E−10	2.10E−18	1.10E−14	12	83	71
PM20D1	cg17178900	7.00E−08	1.20E−19	1.20E−13	-	1.20E−13	4.10E−09	40	87	47

Values represent CpG DNA methylation and SNP correlation Bonferroni-corrected *p* values from Hannon et al. [14], *n* = 166 prefrontal cortex samples (https://epigenetics.essex.ac.uk/mQTL/). *Min* lowest value, *Max* highest value, *Delta* highest − lowest values. Significantly differentially probes for each gene are represented. “-,” not significant

**Table 2:** *PM20D1* mQTL region genes and SNPs and their RNA expression correlations from GTEX and LIBD databases and our own cohort

	rs1172198						rs708727					
	GTEX Hip	GTEX CB	GTEX TN	LIBD PFC	LIBD Hip	*FCX*	GTEX Hip	GTEX CB	GTEX TN	LIBD PFC	LIBD Hip	*FCX*
NUCKS1	n.s.	n.s.	3.30E−12	n.s.	n.s.	*n.s.*	n.s.	1.80E−05	1.00E−11	n.s.	n.s.	*n.s.*
RAB7L1	n.s.	4.00E−08	1.50E−12	n.s.	5.20E−06	*n.s.*	n.s.	8.20E−07	7.80E−13	n.s.	4.40E−03	*n.s.*
SLC41A1	n.s.	3.60E−06	5.00E−08	n.s.	n.s.	*n.s.*	n.s.	2.00E−06	3.20E−10	n.s.	n.s.	*n.s.*
PM20D1	1.40E−07	n.s.	1.90E−36	2.04E−7	1.34E−03	*0.0185*	2.40E−13	n.s.	1.60E−66	4.73E−14	3.83E−07	*2.83E*−*07*
	rs823082						rs823088					
	GTEX Hip	GTEX CB	GTEX TN	LIBD PFC	LIBD Hip	*FCX*	GTEX Hip	GTEX CB	GTEX TN	LIBD PFC	LIBD Hip	*FCX*
NUCKS1	n.s.	n.s.	n.s.	n.s.	n.s.	*n.s.*	n.s.	n.s.	n.s.	n.s.	n.s.	*n.s.*
RAB7L1	n.s.	n.s.	4.30E−10	3.37E−04	6.71E−07	*n.s.*	n.s.	n.s.	n.s.	8.25E−04	2.75E−05	*n.s.*
SLC41A1	n.s.	n.s.	7.40E−09	5.55E−03	n.s.	*n.s.*	n.s.	n.s.	n.s.	4.32E−04	n.s.	*n.s.*
PM20D1	3.20E−09	n.s.	1.40E−29	9.97E−06	0.02515	*3.10E*−*4*	n.s.	n.s.	3.20E−16	0.00279	n.s.	*4.94E*−*04*
	rs1361754						rs960603					
	GTEX Hip	GTEX CB	GTEX TN	LIBD PFC	LIBD Hip	*FCX*	GTEX Hip	GTEX CB	GTEX TN	LIBD PFC	LIBD Hip	*FCX*
NUCKS1	n.s.	n.s.	n.s.	n.s.	n.s.	*n.s.*	n.s.	n.s.	7.60E−06	n.s.	n.s.	*n.s.*
RAB7L1	n.s.	n.s.	4.30E−10	2.61E−04	1.12E−06	*n.s.*	n.s.	n.s.	4.60E−06	n.s.	1.53E−03	*n.s.*
SLC41A1	n.s.	n.s.	7.40E−09	n.s.	n.s.	*0.0477*	n.s.	n.s.	n.s.	n.s.	n.s.	*n.s.*
PM20D1	3.20E−09	n.s.	1.40E−29	2.18E−05	0.02887	*4.85E*−*06*	n.s.	n.s.	3.00E−29	6.25E−06	4.80E−05	*0.0367*

Values in GTEX and LIBD columns represent gene expression and SNP correlation FDR-corrected *p* values from GTEX [12] (https://www.gtexportal.org) and LIBD (http://eqtl.brainseq.org) [13] datasets. GTEX, hippocampus (Hip) *n* = 111, cerebellum (CB) *n* = 154, tibial nerve (TN) *n* = 361; LIBD, dorsolateral prefrontal cortex (PFC) *n* = 412, hippocampus (Hip) *n* = 394. In italics, correlation *p* values obtained from our cohort of frontal cortex samples (*FCX*) *n* = 39. *n.s.* not significant

Hence, we have expanded our previous analysis and measured the levels of DNA methylation of these genes in a genetically well-characterized human cohort of brain samples by locus-specific bisulfite sequencing and pyrosequencing (Fig. 1b, c). We observed no significant correlations between *NUCKS1*, *RAB7L1*, and *SLC41A1* DNA methylation levels and the genetic background (Fig. 1b, c). In fact, *NUCKS1*, *RAB7L1*, and *SLC41A1* promoter regions remained largely unmethylated, independently of the genetic background (Fig. 1b). In contrast, *PM20D1* DNA methylation was strongly correlated with the genetic background (Fig. 1b, c). Thus, despite of a previously reported slight correlation for *SLC41A1*, i.e., values ranging from 7 to 9% of DNA methylation [14] (Table 1), *PM20D1* arises as the only gene in this QTL region that truly qualifies as mQTL.

### RNA expression and genetic background

Similar to DNA methylation, we analyzed the RNA expression levels of the *PM20D1* QTL genes by allele-specific and real-time PCR (Fig. 1d, e). *NUCKS1*, *RAB7L1*, and *SLC41A1* were found to be expressed from both chromosomes (Fig. 1d), and showed no significant correlation with the genetic background, although a trend for *SLC41A1* was observed (Fig. 1e). In contrast, the expression levels of *PM20D1* were significantly correlated with the genetic background, and *PM20D1* was mainly expressed from non-methylated chromosomes (Fig. 1b, c).

**Fig. 2:**
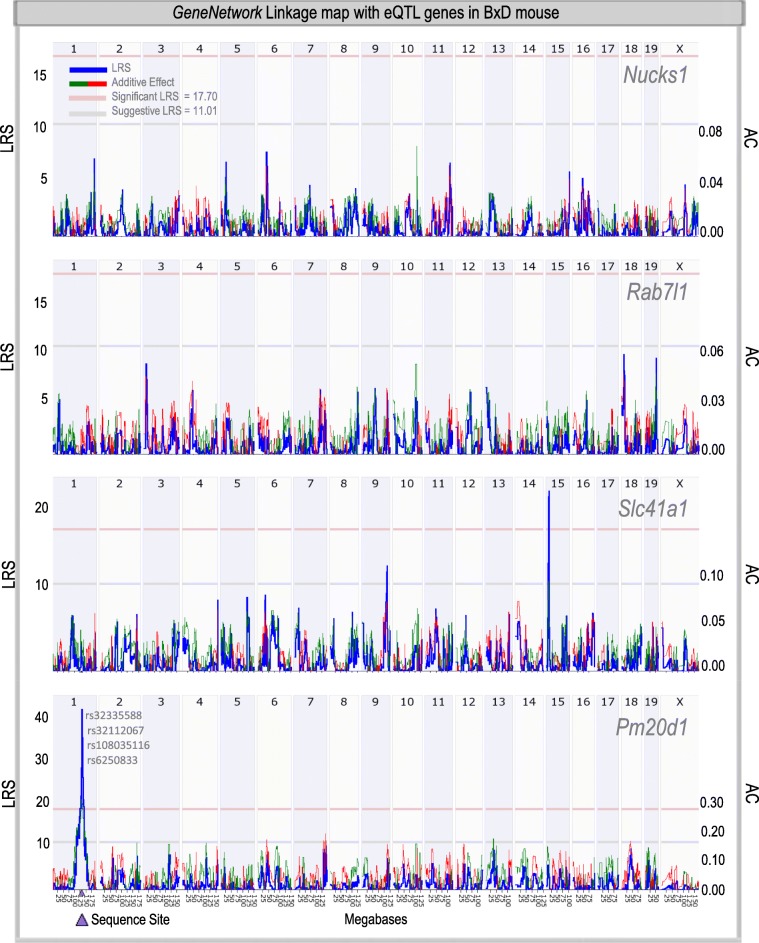
eQTL analysis overview of BxD mouse cohort extracted from the GeneNetwork database. The *PM20D1* QTL region is located on chromosome 1, where the sequence site is indicated (in purple). Top correlated SNPs are indicated in gray. Top values represent chromosomes. Left values represent *likelihood ratio statistics* (LRS), blue line. Thresholds for suggestive and significant LRS are indicated in gray and red, respectively. Right values represent *additive coefficients* (AC), green and red lines for C57BL/6 J (B) and DBA/2 J (D) alleles, respectively

To further explore the *PM20D1* QTL region, we took advantage of the BxD mouse population [15], since mice and humans share a large number of synteny blocks [18], including the *PM20D1* QTL locus. Supporting our previous results, similar relationships were also observed in the BxD population, i.e., no effect of the genetic background for *Nucks1*, *Rab7l1*, and *Slc41a1*, but a strong correlation for *Pm20d1* (Fig. 2). Taken together, these results suggest that in both, human and mice, the strongest eQTL effect is centered on *PM20D1* in the region under investigation.

### QTL expression in Alzheimer’s disease

**Fig. 3:**
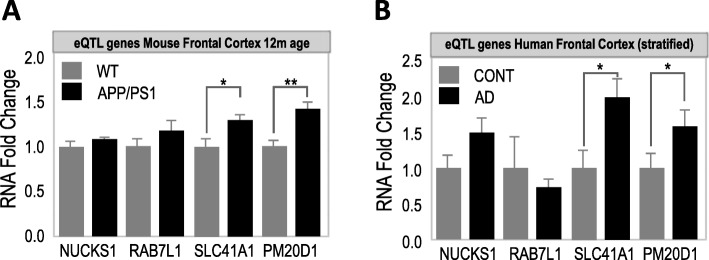
Expression of *PM20D1* QTL genes in AD. **a** Expression of *PM20D1* QTL genes in human AD samples stratified by genotype. **b** Expression of *PM20D1* QTL genes in APP/PS1 frontal cortex samples. Data are presented as means ± SEM. *p < 0.05; **p < 0.01; one-way ANOVA, post hoc Holm-Sidak’s multiple comparison test

Genes in close proximity tend to share common regulatory elements and to correlate in expression [19, 20]. *PM20D1* is upregulated in human and mouse samples of AD [11], which stipulates that its neighboring genes might be dysregulated in similar fashion. Accordingly, both *SLC41A1* and *PM20D1* eQTL low expression carriers seem to be associated with the same AD-risk haplotype (e.g., rs708727 AA carriers, Table 2 and Fig. 1e). To account for this possibility, we assessed the levels of expression of the *PM20D1* QTL genes in APP/PS1 mice and AD human postmortem samples, which were stratified by the rs708727 genotype. No significant expression differences were observed for *NUCKS1* or *RAB7L1* in the frontal cortex of APP/PS1 mice at symptomatic stages (Fig. 3a), nor in human AD frontal cortex (Fig. 3b). Conversely, *SLC41A1* levels were significantly increased in both APP/PS1 mice (Fig. 3a) and human AD samples, together with *PM20D1* (Fig. 3b), which raises the question whether *SLC41A1* might have similar neuroprotective functions as *PM20D1* or, alternatively, whether it participates of AD pathology.

### Functional validation

**Fig. 4:**
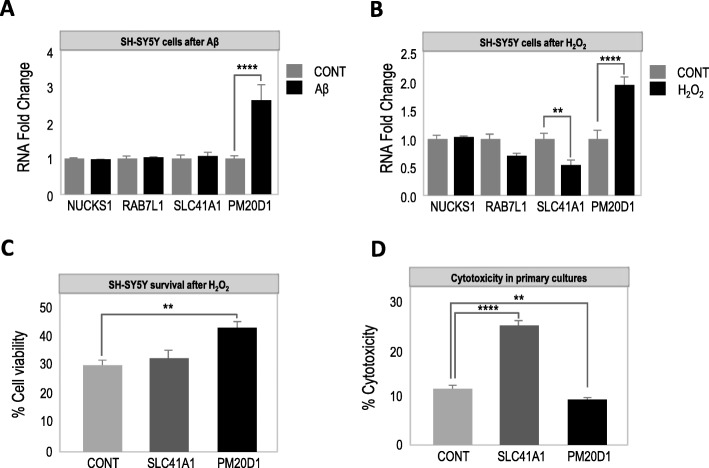
Functional validation of *PM20D1* QTL genes in vitro. **a** *PM20D1* QTL gene expression in SH-SY5Y cells after Aβ treatment. **b** *PM20D1* QTL gene expression in SH-SY5Y cells after H_2_O_2_ treatment. **c** Cell viability of PM20D1 and SLC41A1 overexpressing SH-SY5Y cells after H_2_O_2_ treatment. **d** PM20D1 and SLC41A1 overexpression cytotoxicity in primary cultures. Data are presented as means ± SEM. *p < 0.05; **p < 0.01; ***p < 0.005; ****p < 0.0001; one-way ANOVA, post hoc Holm-Sidak’s multiple comparison test

To further disentangle the potential influence of *PM20D1* QTL genes in AD, we next assayed their expression upon Aβ and ROS exposure, two pathogenic hallmarks of AD [2]. No significant differences were observed for *NUCKS1* or *RAB7L1* genes (Fig. 4a, b). Conversely, both Aβ and ROS treatments significantly increased *PM20D1* (Fig. 4a, b), while ROS but not Aβ altered *SLC41A1* expression, albeit in opposite direction (Fig. 4a, b). Of note, the expression of *PM20D1* and *SLC41A1* was expected to be positively correlated since both eQTLs are associated with the same AD-risk haplotype—i.e., they share common regulatory elements—and both genes are increased in mouse and human samples of AD. However, our results suggest that *PM20D1* and *SLC41A1* are regulated by different mechanisms since only *PM20D1* is upregulated by AD-related stressors—i.e., Aβ and ROS.

Lastly, to investigate whether *SLC41A1*, alongside with *PM20D1* [11], might also influence AD progression, we overexpressed both genes in SH-SY5Y cells and primary neuronal cultures using lentiviral constructs, and assayed both ROS-induced cell death and cell viability. Confirming our previous results [11], PM20D1 overexpression decreased ROS-induced cell death (Fig. 4c) and increased cell viability (Fig. 4d). In contrast, SLC41A1 overexpression did not only not protect against ROS-induced cell death in SH-SY5Y cells (Fig. 4c), but instead proved to be detrimental per se (Fig. 4d), which is reminiscent of a previous report in which SLC41A1 overexpression was shown to reduce cell survival in multiple cell lines [21]. Counterintuitively, the AD-risk haplotype [11] is associated with lower levels of *SLC41A1* expression, together with *PM20D1*, which seems to indicate that the epigenetic association with AD and the upregulation of *SLC41A1* in AD are the consequence of different mechanisms.

In sum, these lines of evidence corroborate our previous results on PM20D1 and suggest that PM20D1—which is upregulated and protective in response to AD-related stressors—is the main gene responsible of the AD-risk haplotype, while the evidence for SLC41A1 is less consistent and in a different direction.

## Discussion

Our previous study has identified *PM20D1* as a risk factor for AD [11]. We found that *PM20D1* DNA methylation and RNA expression were correlated with the genetic background, which, in turn, was associated with AD. Moreover, we demonstrated that by genetically increasing and decreasing PM20D1 expression, AD-related pathologies were decreased and increased, respectively. Recently, the *PM20D1* QTL region has been expanded to other genes, including *NUCKS1*, *RAB7L1*, and *SLC41A1* genes [12, 13] (data shown in Table 2). These genes are in partial linkage disequilibrium with *PM20D1* and, thereby, potentially contribute to our AD-associated risk haplotype.

In order to test this hypothesis, we have performed a comprehensive analysis of *PM20D1* QTL genes, at the DNA methylation, RNA expression, and functional level, using APP/PS1, human postmortem AD samples, as well as mouse in vivo and in vitro experiments. We found no significant correlations between the genetic background and the DNA methylation and/or RNA expression levels of other *PM20D1* QTL genes, except for the previously reported *PM20D1* itself (Fig. 1b–e, Fig. 2, Tables 1 and 2). This is in contrast to other studies, which, in addition to *PM20D1*, reported significant RNA expression correlations with the genetic background for *NUCKS1*, *RAB7L1*, and *SLC41A1 *(Table 2) [12, 13]. However, the strongest effects were found for rs708727 and *PM20D1* DNA methylation, and for rs708727 and *PM20D1* RNA expression in all datasets containing *PM20D1*, which pinpoints PM20D1 as the major QTL in the region (Tables 1 and 2). The reported discrepancies between these studies could lie in the power of the analysis, since the GTEX and LIBD studies contain bigger cohorts; different methods used, i.e., locus-specific versus the high-throughput approaches; and tissue- or brain region-specific eQTL effects, for example, *PM20D1* is not found in the cerebellum GTEX dataset. Consequently, whether *PM20D1* is cell-type and/or tissue-specifically regulated will surely be a matter of future investigations.

Between control and AD samples, we observed no significant differences between the levels of RNA expression of *PM20D1* QTL genes either, except for the previously reported *PM20D1* and the newly reported *SLC41A1*, which were both increased in mouse and human samples of AD (Fig. 3a, b). However, AD-related stressors such as Aβ and ROS exclusively upregulated *PM20D1* expression, but not *SLC41A1*, which was either not affected or downregulated, respectively. In line with these results, only PM20D1 overexpression was found to protect against ROS-mediated cytotoxicity (Fig. 4c) and to increase cell viability (Fig. 4d), whereas SLC41A1 overexpression was either not protective (Fig. 4c) or even detrimental (Fig. 4d). These results are in concordance with other previous investigations. *PM20D1* overexpression and depletion have been shown to be well tolerated [22, 23]. Both adeno-associated virus transduced [22] and PM20D1 knockdown [23] mice are viable and appear healthy, although these manipulations led to altered *N*-acyl amino acid production, which affects thermogenesis regulation [22, 23]. On the contrary, both the overexpression and the depletion of *SLC41A1* have been found to be detrimental. *SLC41A1* overexpression reduced cell survival in multiple cell lines [21], while morpholino-mediated depletion induced severe developmental abnormalities in zebrafish [24], which suggests the need of well-controlled levels of SLC41A1. Interestingly, both *PM20D1* and *SLC41A1* are expressed by astrocytes [25] and might therefore be indirectly associated with increased levels of gliosis in AD [17, 26]. However, as the lack of expression of both PM20D1 and SLC41A1 is associated with a higher risk of AD, but only PM20D1 is upregulated by AD-related stressors and neuroprotective, this suggests that first, PM20D1 is the most relevant gene for AD within the analyzed loci, and second, that the increased levels of SLC41A1 in AD might be a mere consequence of the increased number of astrocytes.

Interestingly, PM20D1 has previously been shown to activate mitochondrial uncoupling [22], which promotes neuronal survival [27] and appears to be protective against neurodegenerative disorders including AD and Parkinson’s disease (PD) [28]. Mitochondrial uncoupling plays important roles in the adaptive responses to bioenergetic and oxidative stressors. It decreases ROS production, prevents mitochondrial calcium accumulation, and regulates protein and substrate mitochondrial import [29]. In addition, PM20D1 itself has also been related to several disorders characterized by high levels of ROS, such as diabetes [22] and obesity [22], and to neurodegenerative diseases with strong mitochondrial alterations, including multiple sclerosis [30] and PD [31]. Taken together, these data suggest a potential use of PM20D1-derived treatment approaches not only for AD, but likely also other diseases.

In sum, our results further support that *PM20D1* is the most likely responsible candidate of the previously reported QTL association with AD, and reinforce its protective role in AD. How precisely PM20D1 exerts its protective functions now requires further investigations.

## Supplementary information

**Additional file 1: Table S1.** List of primers used for the different techniques.

## Data Availability

The datasets generated and analyzed during the current study are not publicly available but are available from the corresponding author on reasonable request.

## Abbreviations

AD: Alzheimer’s disease
eQTL: Expression quantitative trait loci
EWAS: Epigenome-wide association studies
GFAP: Glial fibrillary acidic protein
GWAS: Genome-wide association studies
mQTL: Methylation quantitative trait loci
NUCKS1: Nuclear Casein Kinase And Cyclin Dependent Kinase Substrate 1
PM20D1: Peptidase M20 Domain Containing 1
QTL: Quantitative trait loci
RAB7L1: RAB7, member RAS oncogene family-like 1
ROS: Reactive oxygen species
SLC41A1: Solute Carrier Family 41 Member 1
SNPs: Single nucleotide polymorphisms
